# Quality of Life in Japanese Men Treated with Intensity Modulated Radiotherapy for Localized Prostate Cancer: Three-Year Longitudinal Evaluation Using Patient-Reported Outcomes of the Expanded Prostate Index Composite (EPIC)

**DOI:** 10.3390/jcm15051780

**Published:** 2026-02-26

**Authors:** Norio Mitsuhashi, Atsushi Motegi, Hajime Ikeda, Yoshitaka Nemoto, Daichi Tominaga, Fumiya Shiina, Yukiko Muto, Keiko Fukaya, Atsushi Yamauchi, Shinichi Yoshii

**Affiliations:** 1 Department of Radiation Therapy, Hitachi, Ltd., Hitachinaka General Hospital, Hitachinaka City 312-0057, Japan; 2 Department of Urology, Hitachi, Ltd., Hitachinaka General Hospital, Hitachinaka City 312-0057, Japan

**Keywords:** quality of life, expanded prostate index composite, prostate cancer, intensity modulated radiotherapy, androgen deprivation therapy

## Abstract

**Background/Objectives:** We assessed the changes in the quality of life (QOL) of patients with localized prostate cancer who were treated with IMRT, either with or without Androgen Deprivation Therapy (ADT), using the Expanded Prostate Index Composite (EPIC). **Methods:** Changes in EPIC summary and subdomain scores were evaluated using longitudinal analyses at eight time points up to three years after IMRT. **Results:** The urinary score and four subdomain scores decreased significantly four weeks after the start of IMRT but returned to the baseline level three months after IMRT. This pattern of change remained consistent, regardless of whether ADT was administered or not. The longitudinal changes in bowel score were the same as those in the urinary score. The recovery of the bowel bother subdomain score was rapid, occurring as early as one month after IMRT. Regardless of whether ADT was administered, there was no difference in longitudinal changes in bowel scores. The sexual score remained consistently low throughout the survey period, ranging from 33 to 35. The baseline score for the sexual bother subdomain was 94.44, but the score for the sexual function subdomain was extremely low at 8.24. The hormonal score at the start of IMRT was 87.37 but increased significantly at two and three years after IMRT. The hormonal bother subdomain score decreased significantly six months after IMRT initiation but subsequently increased, becoming significantly higher three years after IMRT. **Conclusions:** IMRT has made it possible to minimize deterioration in the quality of life of patients with localized prostate cancer by reducing adverse events.

## 1. Introduction

As serum PSA testing becomes more widespread, the number of patients with early-stage prostate cancer is increasing. Prostate cancer affected approximately 96,000 Japanese patients in 2021, making it the most prevalent cancer among Japanese men [1].

There are various treatment options available for localized prostate cancer. These include radical prostatectomy; external beam radiation therapy, including 3D-conformal radiotherapy, Intensity Modulated Radiotherapy (IMRT), Intensity Modulated Proton Therapy (IMPT), and carbon beam therapy, brachytherapy, Androgen Deprivation Therapy (ADT), and active surveillance. However, the similarity of treatment outcomes across these diverse therapies can make choosing a treatment challenging for many patients. Therefore, treatment selection for patients with localized prostate cancer should be based on clinical assessments, including disease severity and treatment outcomes such as survival and recurrence rates; adverse events; and health-related quality of life during and after treatment [2,3].

The Expanded Prostate Cancer Index Composite (EPIC) was developed in 2000 by Wei et al. as an expansion of the UCLA Prostate Cancer Index (PCI) to incorporate current treatment methods [4,5]. Although the UCLA PCI scale is already widely used to measure quality of life (QOL) in patients with localized prostate cancer, the EPIC scale was designed to address its perceived shortcomings [5,6]. Compared to the PCI scale, the EPIC scale can assess both irritative and obstructive urinary symptoms and measure the effects of hormone therapy and its associated burden.

Many reports have been published about the QOL of patients with localized prostate cancer who have undergone various treatments, including external radiation therapy [6,7,8,9,10,11]. However, the rapid advancement of radiation therapy technology in recent years has led to the widespread adoption of IMRT in Japan. Consequently, the number of patients with localized prostate cancer who choose radiation therapy has increased significantly. IMRT reduces adverse events, such as rectal, bladder, and urethral complications. Nevertheless, the impact of IMRT on the QOL of these patients has not yet been adequately evaluated.

Therefore, we used the EPIC to assess the QOL of patients with localized prostate cancer who were treated with IMRT, either with or without ADT.

## 2. Materials and Methods

### 2.1. Patient and Tumor Characteristics

A total of 267 consecutive patients with localized prostate cancer who were treated with IMRT with or without ADT at a single institution were enrolled in this study. These patients were referred to our department for IMRT between July 2016 and August 2021 and followed up with our department for at least for three years after IMRT treatment. The Institutional Ethics Review Board approved this study (study number 14-003, BOE-38-001_202506-01, Approved on 26 May 2014). All participants provided written informed consent, and the study protocol was conducted in accordance with the World Medical Association’s Code of Ethics (Declaration of Helsinki).

The mean age ± standard deviation (SD) of the patients was 71.5 ± 5.9 years (range 53~82 years). Table 1 shows the tumor characteristics. Regarding the T factor, 2, 120, 71, 7, 23, 30, and 14 patients had T1b, T1c, T2a, T2b, T2c, T3a, and T3b disease, respectively. Of the 267 patients, 193 (72.3%) had stage I disease.

The most common Gleason score (GS) was 3 + 3, observed in 92 patients. The next most common score was 4 + 3, observed in 56 patients. This was followed by 3 + 4 and 4 + 4, which were each observed in 41 patients. Serum PSA levels of ≤5, 5~10, 10~20, and ≥20 ng/mL were present in 20, 127, 72, and 48 patients, respectively. According to the D’Amico clinical risk classification, 64, 93, and 110 patients were classified low, intermediate, and 110 as high risk, respectively.

All patients were pathologically classified as adenocarcinomas, except for one patient with neuroendocrine cancer.

### 2.2. Instruments for the QOL Assessment

The EPIC is the most widely used QOL assessment tool for patients with localized prostate cancer. It has been translated into over 170 languages and is used internationally [12,13,14,15,16]. A Japanese version is available and linked to the Japanese version of the SF-8 [6,17]. This study used the Japanese version of the EPIC to proxy for disease-specific QOL [5,6,7]. The EPIC is a 50-item questionnaire that quantifies prostate cancer-specific QOL in four domains: urinary, bowel, sexual, and hormonal. Each domain contains function and bother subscales. Total scores can be calculated for each domain and each subscale. EPIC QOL scores from all domains were linearly transformed onto a scale from 0 (lowest) to 100 (highest). Higher domain scores represent better functioning and QOL.

We evaluated changes and recovery patterns for patients over time using longitudinal approaches. We examined mean scores at the start of IMRT (baseline), four weeks after initiating IMRT (at 40 Gy in 20 fractions), and one, three, and six months and one, two, and three years after completing IMRT.

Additionally, the effect of ADT on EPIC QOL scores was analyzed by comparing the mean EPIC QOL scores across the three domains (excluding the hormonal domain) and their subdomains between ADT-treated patients and non-ADT-treated patients.

### 2.3. Treatment

#### 2.3.1. Androgen Deprivation Therapy (ADT)

Table 2 shows the treatment characteristics of the patients. According to the risk classification, ADT was administered as follows: no ADT for patients in the low-risk group; 4~6 months of neoadjuvant ADT for those in the intermediate-risk group; and 4~6 months of neoadjuvant ADT and 24~36 months of adjuvant ADT for those in the high-risk group. However, in the first half of this study, patients in the group with a GS of 3 + 4 were treated with IMRT alone, without ADT. ADT mainly comprised the administration of a luteinizing hormone-releasing hormone agonist plus a nonsteroidal or steroidal anti-androgen. A total of 187 patients (70.0%) received ADT in combination with IMRT. Of these patients, 104 (55.6%) received ADT for more than one year (the long-term ADT group) and 83 patients (44.4%) received ADT for less than one year (the short-term ADT group). The mean age ± SD was 69.1 ± 6.0 years (range: 54~78 years) for the patients treated with IMRT alone. The mean age was 72.6 ± 5.6 years (range: 53~82 years) for patients who received ADT in combination with IMRT. There was a statistically significant difference in mean age between the two groups (*p* < 0.001).

#### 2.3.2. Intensity Modulated Radiotherapy (IMRT)

As shown in Table 2, the total radiation dose was 74 Gy in 37 fractions. IMRT was performed daily. One patient with radioresistant neuroendocrine cancer received 69 Gy in 23 fractions every other day. For six patients in the highest-risk group in the latest period of this study, the dose was increased to 78 Gy in 39 fractions. IMRT was completed as scheduled for all patients except two: one in whom another cancer was detected and one who refused treatment. Two radiation oncologists delineated the planning target volumes and organs at risk (OARs) on CT slices using CT images superimposed with T2-MR images and the contouring tool of the Pinnacle^3^ treatment planning system v9.10 (Philips Healthcare, Fitchburg, WI, USA).

Various volumes of interest were defined: The GTV (prostate), the CTV (prostate and proximal seminal vesicle), and the PTV (CTV +8 mm margin, except for a 5 mm margin posteriorly) were outlined, as were the OARs. The rectum, bladder, penile bulb, and both femoral heads were outlined. The bladder and rectum were defined by contouring the entire organ and its contents. After simulation and contouring, the target volumes were planned using the Convolution Superposition Algorithm in Pinanacle^3^. All plans used 10 MV photons. The dose was delivered using volumetric modulated arc therapy (VMAT), a type of IMRT.

From two weeks before the initiation of IMRT throughout the treatment period, laxatives were administered to eliminate gas and feces in the rectum and keep the prostate in a constant position. This was confirmed using cone beam CT at the start of each session. Additionally, ultrasound was used to confirm the presence of 150–200 mL of urine in the bladder before each irradiation session began to maintain a constant size.

### 2.4. Treatment Outcomes

During the three-year observation period, PSA failure was the most common cause of recurrence (16 patients, or 4.0%), followed by bone metastasis (four patients, or 1.6%), lymph node metastasis (one patient, or 0.4%), and lung metastasis (one patient, or 0.4%).

According to the RTOG and EORTC toxicity criteria [18], there were no grade 2 or higher acute genitourinary or gastrointestinal toxicities. Late toxicities are shown in Table 3. Only one patient developed grade 3 genitourinary toxicity.

### 2.5. Statistical Analysis

We used unpaired, two-tailed *t*-tests to analyze the mean scores in the four domains and their respective subdomains at each observation time point.

We set the level of statistical significance at *p* < 0.05 for all statistical analyses. All analyses were performed using Microsoft^®^ Excel^®^ for Windows, version 16.26 (Microsoft Corporation, Redmond, WA, USA).

## 3. Results

### 3.1. Urinary Scores

#### 3.1.1. All Patients

**Figure 1:**
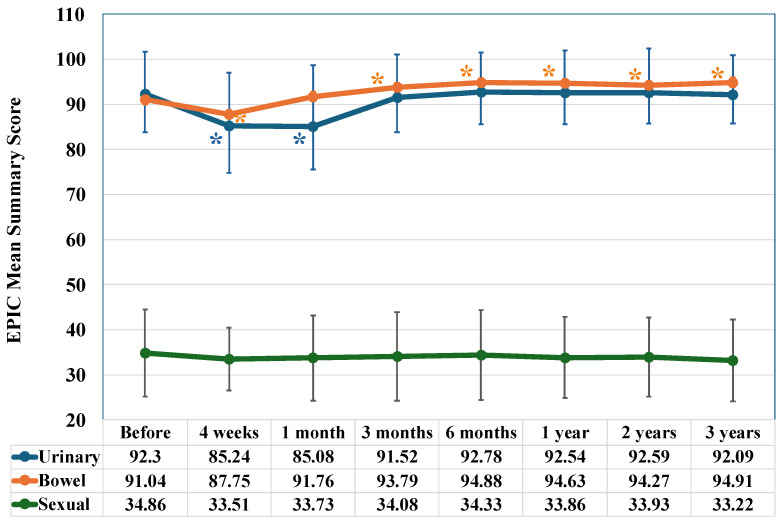
Longitudinal changes in EPIC mean urinary, bowel, and sexual summary scores. Evaluation at the time of initiating IMRT, then 4 weeks after initiating IMRT (40 Gy/20 fractions), and again at 1, 3, and 6 months and 1, 2, and 3 years after completing IMRT. An asterisk (*) below the curve indicates a significant decrease in score compared to the baseline, where an asterisk above the curve indicates a significant increase.

The urinary score decreased significantly four weeks after the start of IMRT (*p* < 0.001) and one month after its completion (*p* < 0.001), but returned to baseline levels after three months (Figure 1).

**Figure 2:**
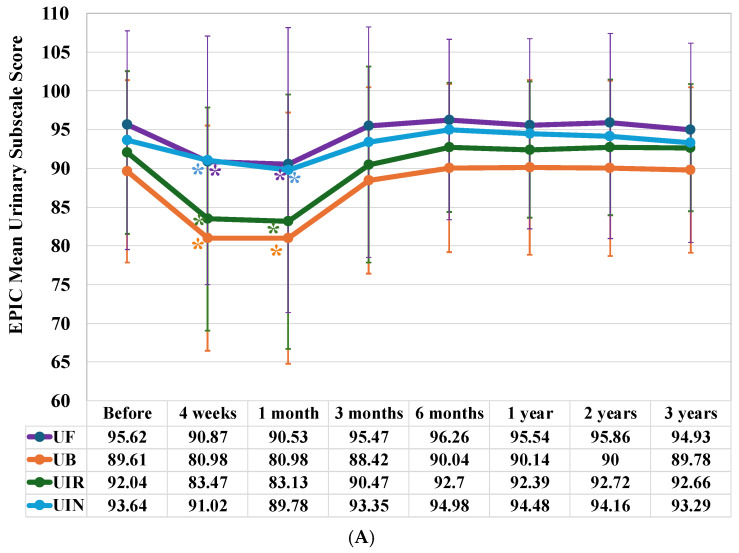

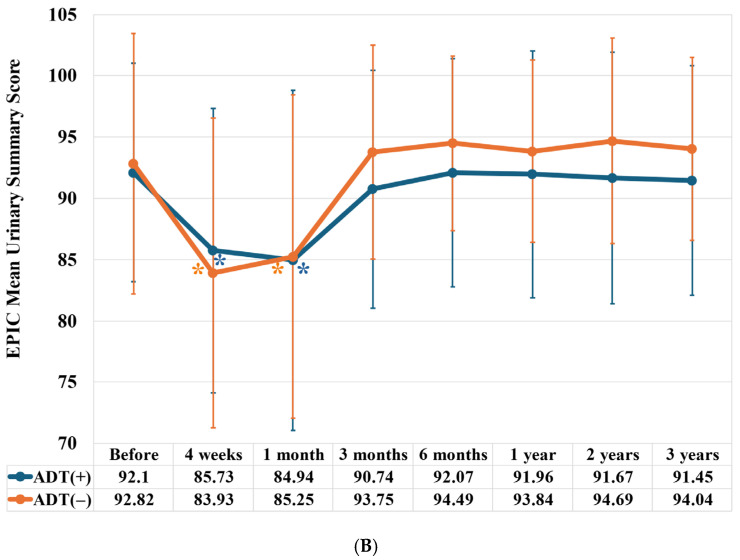
Longitudinal changes in EPIC mean urinary summary and subscale scores. (**A**) Longitudinal changes in EPIC mean urinary subscale scores. UF: urinary function, UB: urinary bother, UIR: urinary irritative obstruction, and UIN: urinary incontinence. (**B**) A comparison of longitudinal changes in EPIC mean urinary summary scores between patients treated with Intensity Modulated Radiotherapy (IMRT) alone and those treated with IMRT combined with Androgen Deprivation Therapy (ADT). The evaluation timeline is the same as in Figure 1. An asterisk (*) below the curve indicates a significant decrease in score compared to the baseline, where an asterisk above the curve indicates a significant increase.

Examining urinary scores by subdomain revealed significant decreases in all domains: urinary function (UF) (at four weeks and one mouth, *p* < 0.001), urinary bother (UB) (at four weeks and one month, *p* < 0.001), urinary irritative obstruction (UIR) (at four weeks and one month, *p* < 0.001), and urinary incontinence (UIN) (at four weeks, *p* = 0.048 and one month, *p* = 0.008). These decreases occurred up to one month after completing IMRT (Figure 2A). However, all domains subsequently recovered to baseline levels.

#### 3.1.2. Patients Treated with ADT

The urinary score decreased significantly four weeks after the initiation of IMRT (*p* < 0.001), and one month after its completion (*p* < 0.001). However, it returned to baseline levels after three months (Figure 2B).

#### 3.1.3. Patients Treated Without ADT

The urinary score decreased significantly four weeks after the initiation of IMRT (*p* < 0.001) and one month after its completion (*p* = 0.003). However, it returned to baseline levels after three months (Figure 2B).

### 3.2. Bowel Scores

#### 3.2.1. All Patients

The bowel score decreased significantly four weeks after the start of IMRT (*p* < 0.001), as shown in Figure 1. However, it began to recover one month after IMRT, increasing significantly (*p* < 0.001). This increase persisted for three years (at six months, *p* < 0.001; at one year, *p* < 0.001; at two years, *p* < 0.001; and at three years, *p* < 0.001).

The bowel function (BF) subdomain score decreased significantly four weeks after the start of IMRT (*p* < 0.001) but increased significantly over the following three years after IMRT (at one month, *p* = 0.039; at three months, *p* < 0.001; at six months, *p* < 0.001; at one year, *p* < 0.001; at two years, *p* < 0.001; and at three years, *p* < 0.001).

**Figure 3:**
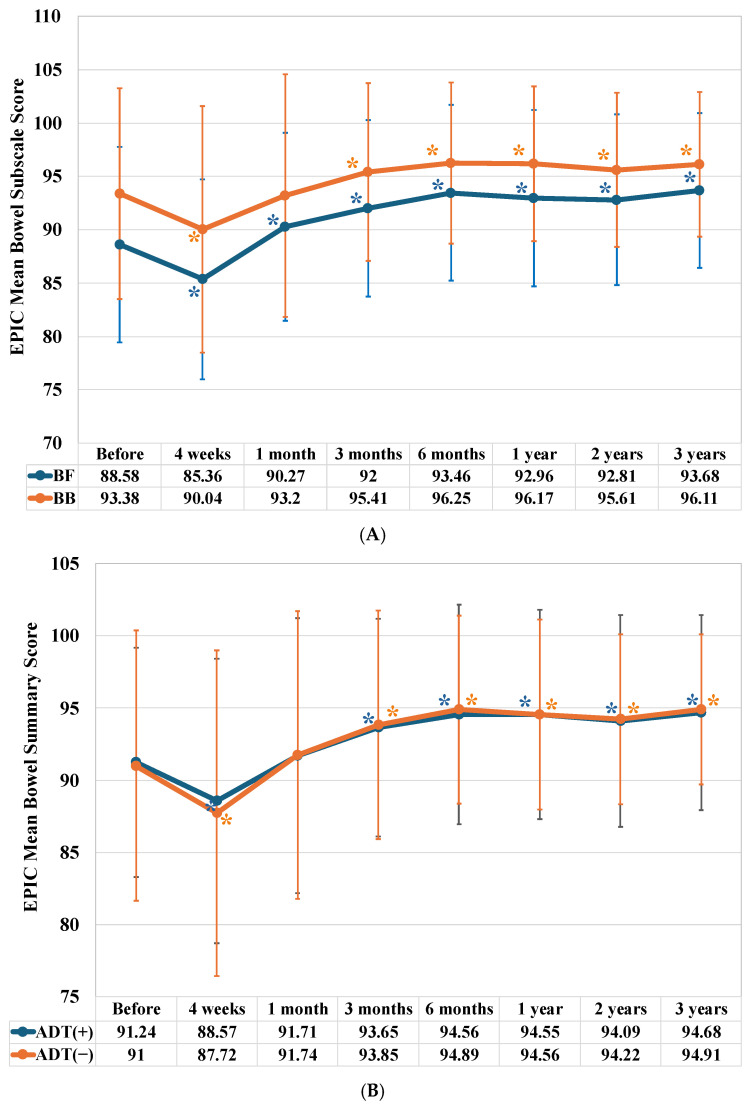
Longitudinal changes in EPIC mean bowel summary and subscale scores. (**A**) Longitudinal changes in EPIC mean urinary subscale scores. BF: bowel function and BB: bowel bother. (**B**) A comparison of longitudinal changes in EPIC mean bowel summary scores between patients treated with Intensity Modulated Radiotherapy (IMRT) alone and those treated with IMRT combined with Androgen Deprivation Therapy (ADT). The evaluation timeline is the same as in Figure 1. An asterisk (*) below the curve indicates a significant decrease in score compared to the baseline, where an asterisk above the curve indicates a significant increase.

The bowel bother (BB) subdomain score also decreased significantly four weeks after the start of IMRT (*p* < 0.001) and increased significantly three months (*p* = 0.013) after IMRT completion. It remained high for the following three years: six months (*p* < 0.001), one year (*p* < 0.001), two years (*p* = 0.006), and at three years (*p* < 0.001). This pattern was similar to that of the BF subdomain (Figure 3A).

#### 3.2.2. Patients Treated with ADT

The bowel score decreased significantly four weeks after the start of IMRT (*p* = 0.0046), but then increased gradually. A significant increase compared with the baseline score was observed three months after the end of IMRT (*p* = 0.00036), as well as at six months (*p* < 0.001), one year (*p* < 0.001), two years (*p* = 0.010), and three years (*p* < 0.001) (Figure 3B).

#### 3.2.3. Patients Treated Without ADT

The bowel score decreased significantly four weeks after the start of IMRT (*p* = 0.031), but then increased gradually. A significant increase was observed three months after the end of radiation therapy compared to the baseline score: This increase was significant at three months (*p* = 0.023), six months (*p* = 0.0012), one year (*p* = 0.0056), two years (*p* = 0.0056), and three years (*p* = 0.0087) (Figure 3B). Regardless of whether ADT was administered, there was no difference in the longitudinal changes in mean bowel scores.

### 3.3. Sexual Score

#### 3.3.1. All Patients

The sexual score remained consistently low, ranging from 33 to 35, from the start of treatment through the survey period. The sexual score showed a slight decrease with significant trends observed four weeks (*p* = 0.07) after the start of IMR and three years after its completion, respectively, though no significant change was observed (Figure 1).

**Figure 4:**
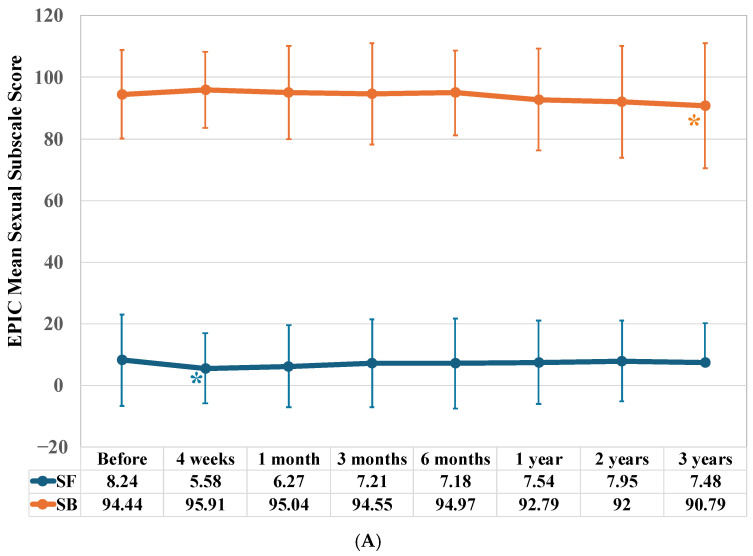

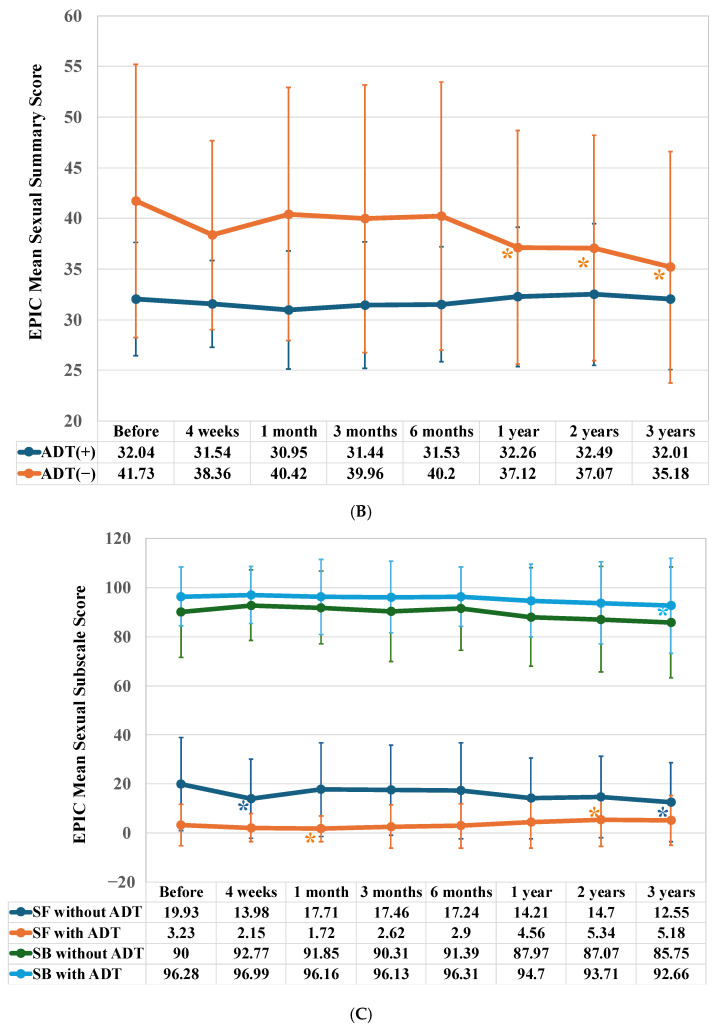
Longitudinal changes in EPIC mean sexual summary and subscale scores. (**A**) Longitudinal changes in EPIC mean sexual subscale scores. (**B**) A comparison of longitudinal changes in EPIC mean sexual summary scores between patients treated with Intensity Modulated Radiotherapy (IMRT) alone and those treated with IMRT combined with Androgen Deprivation Therapy (ADT). (**C**) A comparison of longitudinal changes in EPIC mean sexual subscale scores between patients treated with Intensity Modulated Radiotherapy (IMRT) alone and those treated with IMRT combined with Androgen Deprivation Therapy (ADT). SF: sexual function, SB: bowel bother. The evaluation timeline is the same as in Figure 1. An asterisk (*) below the curve indicates a significant decrease in score compared to the baseline, where an asterisk above the curve indicates a significant increase.

At the start of IMRT, the baseline sexual bother (SB) subdomain score was 94.4 and showed only a slight decrease. Meanwhile, the sexual function (SF) subdomain score was an extremely low 8.24. The SF subdomain score decreased significantly four weeks after the start of IMRT (*p* = 0.022) (Figure 4A). Although it increased thereafter, it did not reach baseline levels. However, the difference was not statistically significant. The sexual bother (SB) subdomain score only decreased significantly compared to the baseline score after three years of IMRT (*p* = 0.025).

#### 3.3.2. Patients Treated with ADT

The sexual score remained at approximately 31 throughout the three-year study, with no significant changes observed compared to the baseline score. However, the baseline score was significantly lower than that of patients treated without ADT (*p* < 0.001) (Figure 4B).

Borderline scores for both the SF and SB subdomains were significantly lower than those for patients not treated with ADT (SF: *p* < 0.001; SB: *p* = 0.002) (Figure 4C).

The SF subdomain score decreased significantly one month after IMRT completion (*p* = 0.044). Then, it began to increase, showing a significant rise two years after IMRT completion (*p* = 0.0043). The SB subdomain score showed no significant change up to two years after IMRT completion. However, it decreased significantly three years after IMRT completion (*p* = 0.037).

#### 3.3.3. Patients Treated Without ADT

The baseline sexual score was significantly higher for patients treated with IMRT alone (41.73) than for those treated with IMRT combined with ADT. A significant increase in the score was observed one year or more after completing IMRT, compared to the baseline score (at one year, *p* = 0.033; at two years, *p* = 0.038; and at three years, *p* = 0.0041).

The SF subdomain score decreased significantly four weeks after completing IMRT (*p* = 0.047). It then began to increase but did not return to baseline levels. The score declined again one year after treatment completion and remained significantly lower than the baseline score three years after treatment completion (*p* = 0.021). However, there were no significant changes in the SB subdomain score during the observation period.

### 3.4. Hormonal Score

Self-report questionnaires to evaluate the hormonal domain score and the two hormonal subdomain scores were only prepared for patients who received ADT. Therefore, patients treated with IMRT alone did not answer these questions.

**Figure 5:**
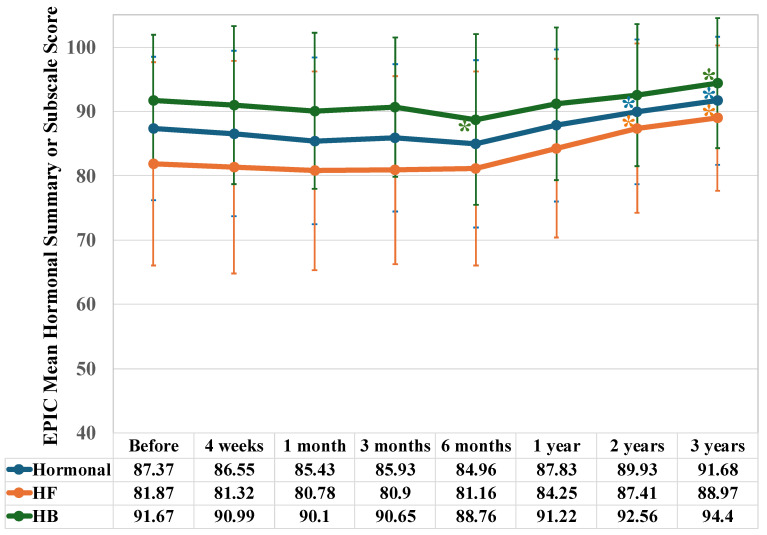
Longitudinal changes in EPIC: mean hormonal summary and subscale scores. Hormonal: summary score, HF: hormonal function, and HB: hormonal bother. The evaluation timeline is the same as in Figure 1. An asterisk (*) below the curve indicates a significant decrease in score compared to the baseline, where an asterisk above the curve indicates a significant increase.

The hormonal score decreased to 87.4 at the start of IMRT and then increased significantly at the two- and three-years marks (at two years, *p* = 0.039; at three years, *p* < 0.001) (Figure 5).

Scores for the hormonal function (HF) subdomain increased significantly compared to the baseline score two years after IMRT and beyond (at two years, *p* < 0.001; at three years, *p* < 0.001). The hormonal bother (HB) subdomain score decreased after the initiation of IMRT and showed a significant decline six months after its completion (*p* = 0.020). However, it then began to increase, showing a significant rise three years later (*p* = 0.017).

## 4. Discussion

### 4.1. A Comparison of Changes in EPIC Scores Between Patients Who Underwent Radical Prostatectomy and Those Who Received Radiation Therapy

Reports evaluating changes in QOL using the EPIC questionnaire after treatment for localized prostate cancer with different radiation therapy techniques, such as three-dimensional conformal radiation therapy (3D-CRT), IMRT, hypo-fractionated radiation therapy, stereotactic body radiation therapy (SBRT), brachytherapy, and proton therapy, have already been published [7,10,11,19,20,21,22,23,24,25].

Several studies in the literature have compared changes in QOL following radical prostatectomy and radiation therapy using the EPIC questionnaire [7,9,15,17,19]. Ettala et al. reported statistically significant differences in pre- and post-treatment scores between patients who underwent radical prostatectomy or radiation therapy [14]. Radical prostatectomy was associated with an increased risk of worsening urinary incontinence compared to radiation therapy [8]. In their review, Lee et al. concluded that patients with localized prostate cancer who underwent radical prostatectomy experienced different QOL changes compared to those who received radiation therapy. Post-radiation bowel side effect outcomes are less favorable than those of radical prostatectomy, with a low probability of returning to baseline. The overall impact on urinary quality of life is similar for surgical and radiation treatment. However, urinary incontinence is most prevalent after radical prostatectomy and urinary irritative symptoms are most prevalent in patients treated with radiation therapy. On average, brachytherapy was associated with the least impact on sexual QOL and function [7]. In addition, Matsukawa et al. reported that the impact of radiotherapy, including proton beam therapy, on urinary conditions and sexual function was lower than that of surgery in Japanese men [24]. Wilkins et al. reported that the incidence of patient-reported bowel symptoms was low and similar between patients in the 74 Gy control group and the hypo-fractionated group (60 Gy in 20 fractions or 57 Gy in 19 fractions) up to 24 months after radiotherapy [22]. Regarding SBRT, Katz et al. and Van As et al. reported that SBRT at a dose in the range of 35–36.25 Gy in five fractions was associated with a lower incidence of urinary incontinence and sexual dysfunction and a slightly higher incidence of bowel bother than prostatectomy [10,19]. We have reported that long-term QOL scores for the urinary domain and its subdomains may be affected by the high-dose area of the prostate gland (V150) or the urethra (D10) in patients treated with high-dose-rate brachytherapy combined with external beam radiotherapy [23].

### 4.2. A Comparison of the Current Study with Previously Reported Studies on Changes in EPIC Scores Among Patients Treated with IMRT

#### 4.2.1. Changes in Urinary Domain and Subdomain Scores

The current study found a significant decrease in the urinary summary score including in the four subdomains (UF, UB, UIN, and UIR), up to one month after IMRT. This is considered an acute radiation morbidity of the cervix of urinary bladder and the ureter. However, three months after IMRT, the urinary score returned to a borderline level. These results suggest that radiation-induced acute urinary tract disorders improve without delay. This indicates that IMRT is safe for the urinary tract. The recovery process for urinary summary scores in cases of radiation-induced acute urinary tract dysfunction is the same, regardless of whether ADT is administered. This clearly demonstrates that ADT has no effect on radiation-induced acute urinary tract dysfunction or recovery from urinary damage. Similar to our results, Nakai et al. reported that the urinary domain worsened halfway through IMRT, worsened further and more severely immediately after IMRT, and then improved [21]. On the other hand, Yamamoto et al. reported that IMRT did not affect urinary function, including irritative and obstructive symptoms [20].

#### 4.2.2. Changes in Bowel Domain and Subdomain Scores

Our study demonstrated that, although there was a significant decrease in the bowel summary score at four weeks after the initiation of IMRT, including the two subdomain scores (BF and BB), there was a significant increase in the score thereafter. The significant increase in the bowel summary score three months after IMRT occurred because the baseline score had already decreased due to laxatives administered from two weeks prior to the initiation of IMRT throughout the treatment period. These laxatives were used to eliminate gas and stool in the rectum and to maintain the prostate’s position. In other words, the scores during the laxative administration period, including the borderline scores, might have been higher if laxatives had not been administered. Therefore, rather than scores increasing significantly three months after completing IMRT, it is conceivable that they would return to baseline scores for patients not receiving laxatives.

Stenmark et al. revealed that the inferior rectum was associated with a decline in bowel QOL using segmental dose volume histogram analysis [26]. Bulman et al. reported that rectal doses, specifically a biologically effective dose (BED) of D25 (Gy) ≥ 23%, are significantly associated with a decline in bowel bother-related QOL in patients undergoing definitive proton therapy for localized prostate cancer. This study demonstrates that a BED is an independent predictor of bowel QOL across different radiation doses [27].

#### 4.2.3. Changes in Sexual Domain and Subdomain Scores

Patients receiving ADT typically have a significantly lower baseline sexual score at the start of IMRT than patients not receiving ADT. For these patients, the baseline sexual score should be measured at the start of ADT. However, this study did not assess sexual scores at the beginning of ADT treatment. Furthermore, the mean age of patients treated with IMRT and ADT was 72.6 years, contrasting with the significantly younger mean age of 69.1 years in patients treated with IMRT alone. This age difference likely contributed to the lower baseline sexual scores observed in patients treated with IMRT combined with ADT.

Additionally, the low sexual scores observed in patients not receiving ADT can be attributed to their older mean age of 69.1 years, which may have caused a significant decline in mean sexual score one year after IMRT.

Of the 187 patients who received ADT, 83 finished the treatment within one year, suggesting that the effects of hormone therapy diminish over time. Nevertheless, the overall sexual score for all patients receiving ADT did not change from baseline. However, this study cannot determine whether these findings indicate residual effects of hormone therapy persisting beyond two years or whether they are due to aging, as testosterone levels were not measured. Iwamoto et al. reported that total testosterone levels in Japanese men decline by approximately 80% by age 50 years and then remain stable. Free testosterone levels, however, decreased almost linearly to approximately 56% by age 70 years [28]. Nakai et al. pointed out that the sexual domain of the EPIC decreased significantly at the halfway point of IMRT, and continued to decrease until 24 months [21]. Sexual declines are common with radiation therapy, and multiple reports suggest that the penile bulb (PB) may act as a dosimetric surrogate for radiation-associated erectile dysfunction (ED). The QUANTEC analysis identified a mean PB dose of 50 Gy as being associated with ED [29,30]. Seymour et al. reported that the use of a hydrogel spacer was associated with an improved sexual QOL, fewer cases of measurable decline in sexual QOL, and higher rates of adequate erectile function by reducing the radiation dose to the PB [31].

#### 4.2.4. Changes in Hormonal Domain and Subdomain Scores

The decrease in the borderline of the hormonal score in the current study, as well as the HF and HB subdomain scores, was due to four to six months of neoadjuvant ADT. It is considered that the increase in hormone levels over time is due to the fact ADT ended at the beginning of IMRT for the intermediate-risk group and approximately two and a half years after the end of IMRT in the high-risk group. Since ADT was initiated with four to six months of neoadjuvant ADT, IMRT did not delay recovery. Yamamoto et al. also reported that IMRT did not affect hormonal function [20].

#### 4.2.5. Issues with the QOL Assessment Using the EPIC

The EPIC questionnaire is a well-designed, widely used tool for assessing changes in the QOL of prostate cancer patients. However, some reports have pointed out several issues with it. Talvitie et al. point out that patients are generally willing to report their symptoms; however, a lack of suitable response options leads to missing data and inconsistent response strategies in the sexual and urinary domains, which weakens the quality of the data received [32]. Bonet et al. caution that conventional methods of assessing toxicity following radiation therapy may overlook intestinal symptoms, such as urgency. This highlights the need to re-evaluate methods of assessing intestinal toxicity in order to identify patients who are experiencing symptoms that impact their QOL [33]. Roth et al. reported that impaired pretherapeutic self-reported functional status was associated with lower educational level and poorer disease characteristics, except for urinary incontinence, which was only associated with age [34]. Ettala et al. also found that older patients were more likely to leave questions unanswered, especially those questions with multiple items [14].

Most previously reported QOL assessments have examined changes in EPIC scores during the short-term period following treatment. However, considering the potential for late adverse events following treatment, particularly radiation therapy, it is important to monitor long-term changes in QOL. Fossa et al. reported that health professionals should be aware of persisting moderate to severe urinary, bowel, or sexual problems in long-term prostate cancer survivors, as these problems are associated with reduced QOL [35].

## 5. Conclusions

The urinary score, which includes four subdomain scores, and the bowel score, decreased significantly four weeks after the start of IMRT. However, these scores returned to baseline levels three months after IMRT, regardless of whether ADT was administered or not. The BB subdomain score recovered rapidly, improving as early as one month after IMRT. The sexual score remained consistently low throughout the survey period, ranging from 33 to 35. The baseline score for the SB subdomain was 94.44; however, the score for the SF subdomain was extremely low at 8.24. The hormonal score at the start of IMRT was 87.37, but it increased significantly at two and three years after IMRT. The HB subdomain score decreased significantly six months after the initiation of IMRT but subsequently increased, becoming significantly higher three years after IMRT.

IMRT has made it possible to minimize the deterioration of patients’ QOL with localized prostate cancer by reducing adverse events.

## Figures and Tables

**Table 1:** Tumor characteristics of patients with localized prostate cancer.

Category	Item	No of Patients	%
T Factor	T1b	2	0.8
	T1c	120	44.9
	T2a	71	26.6
	T2b	7	2.6
	T2c	23	8.6
	T3a	30	11.2
	T3b	14	5.3
Clinical Stage	I	193	72.3
	II	30	11.2
	III	44	16.5
Gleason Score	3 + 3	92	34.5
	3 + 4	41	15.3
	3 + 5	4	1.5
	4 + 3	56	21
	4 + 4	41	15.3
	4 + 5	28	10.5
	5 + 4	5	1.9
Serum PSA value (ng/mL)	PSA < 5	20	7.5
	5 ≤ PSA < 10	127	47.5
	10 ≤ PSA < 20	72	27
	PSA ≥ 20	48	18
D’Amico clinical risk	Low Risk	64	24
	Intermediate Risk	93	34.8
	High Risk	110	41.2
Pathology	Adenocarcinoma	266	99.6
	Neuroendocrine Cancer	1	0.4

**Table 2:** Treatment characteristics of patients with localized prostate cancer.

Category	Item	No. of Patients	%
Androgen Deprivation Therapy (ADT)	No	80	30.0
	Yes	187	70.0
Duration of ADT treatment	Less than 1 year	83	44.4
	More than 1 year	104	55.6
Total dose/fractions in IMRT	6 Gy/3 Fr	1	0.4
	16 Gy/8 Fr	1	0.4
	69 Gy/23 Fr	1	0.4
	70 Gy/35 Fr	1	0.4
	74 Gy/37 Fr	257	96.3
	78 Gy/39 Fr	6	2.2
Loss of follow-up	Yes	5	1.9
Discontinued radiation therapy	Yes	2	0.7

**Table 3:** Late toxicity according to RTOG/EORTC criteria.

Category	Grade	No. of Patients	%
Genitourinary	Grade_1	16	6.0
	Grade_2	2	0.7
	Grade_3	1	0.4
Gastrointestinal	Grade_1	14	5.2
	Grade_2	2	0.8

## Data Availability

The raw data supporting the conclusions of this article will be made available by the authors on request.

## Abbreviations

The following abbreviations are used in this manuscript:

IMRT	Intensity Modulated Radiotherapy
EPIC	Expanded Prostate Index Composite
QOL	Quality of life
ADT	Androgen Deprivation Therapy
PCI	Prostatic Cancer Index
PSA	Prostate specific antigen
SD	Standard deviation
GS	Gleason score
MR	Magnetic resonance
OARs	Organs at risk
GTV	Gross target volume
CTV	Clinical target volume
VMAT	Volumetric modulated arc therapy
CT	Computed tomography
MV	Megavolt
RTOG	Radiation Therapy Oncology Group
EORTC	European Organization for Research and Treatment of Cancer
UF	Urinary function
UB	Urinary bother
UIN	Urinary incontinence
UIR	Urinary irritative/obstructive
BF	Bowel function
BB	Bowel bother
SF	Sexual function
SB	Sexual bother
HF	Hormonal function
HB	Hormonal bother
SF-8	Short form-8
3D-CRT	Three-dimensional conformal radiation therapy
SBRT	Stereotactic body radiation therapy
V150	Volume receiving 20 Gy
D10	Percentage of organ volume receiving at least 10 Gy
BED	Biologically effective dose
D25	Percentage of organ volume receiving at least 25 Gy
Gy	Gray
PB	Penile bulb
ED	Erectile dysfunction
QUATEC	The Quantitative Analyses Of Normal Tissue Effects In The Clinic
